# Symptom tracking made simple? Observational data on the clinical use of the PERS^2^ON score in cancer patients receiving palliative care

**DOI:** 10.1007/s00508-025-02553-3

**Published:** 2025-05-31

**Authors:** Katharina Tscherny, Juergen Grafeneder, Bettina Wandl, Maximilian Niederer, Martina Haider, Eva Katharina Masel, Dominik Roth, Alexander Egger

**Affiliations:** 1 Department of Anesthesiology and Intensive Care Medicine, Hospital Scheibbs, Eisenwurzenstraße 26, 3270 Scheibbs, Austria; 2 Department of Emergency Medicine, Medical University of Vienna, Waehringer Guertel 18–20, 1090 Vienna, Austria; 3 Department of Medicine I, Division of Palliative Medicine, Medical University of Vienna, Vienna, Austria

**Keywords:** Palliative care, Cancer, Symptom assessment, Self-assessment questionnaire, End-of-life care

## Abstract

**Context:**

A standardized assessment of symptoms is essential for individualized palliative care (PC). While numerous tools exist, many are too complex for daily clinical use.

**Objective:**

The PERS^2^ON score was developed as a brief and practical tool to assess symptom burden in PC. This study aimed to evaluate its feasibility in a rural hospital setting, although it did not include formal feasibility metrics such as recruitment or adherence rates.

**Methods:**

Patients admitted to the palliative care unit of a rural hospital were assessed using the PERS^2^ON score, which includes seven items: pain, eating, rehabilitation, social situation, suffering, oxygen/dyspnea, and nausea/emesis. Each item is scored from 0 to 10, with higher scores indicating greater burden. Assessments were conducted on admission, after 7 days, and at discharge. Symptom scores were analyzed for change over time.

**Results:**

Of 60 admitted patients, 40 met the inclusion criteria. Reassessment was possible in 35 patients after 7 days and in 31 patients at discharge. The mean PERS^2^ON score decreased from 28 (SD 12) on admission to 21 (SD 11) after 7 days (difference: 7, 95% confidence interval, CI: 3–11, *p* = 0.002), and to 17 (SD 10) at discharge (difference: 11, 95% CI: 6–15, *p* < 0.001).

**Conclusion:**

The PERS^2^ON score was feasible to implement and enabled consistent symptom monitoring. Its use may support structured clinical assessment and targeted symptom management even in smaller or rural palliative care units.

## Introduction

Malignancy is currently the second most common cause of death in Austria after cardiovascular disease. Every year, more than 40,000 people are diagnosed with cancer [1]. According to the current data report of the organization *Federation for Hospice and Palliative Care*, 7962 patients were treated in 43 palliative wards and palliative care facilities across Austria in 2020. Of these 72% suffered from a malignancy [2].

Quality of life (QoL) has become an increasingly more medically important concept over the last few decades; however, there is no uniform definition and no generally accepted assessment to measure QoL. Health-related QoL is a subjective perception of mental, physical, spiritual, social, family, and work-related dimensions. Measuring instruments for self-assessment of the QoL of patients enable medical and therapeutic successes to be assessed. These have thus developed into important tools for successful patient management [3, 4].

The assessment of symptoms in patients who are receiving best supportive care (BSC) is a challenging task for clinicians. Traditional clinical assessment often misses target symptoms and can underestimate significant problems in patients undergoing palliative care (PC) [5, 6]; however, therapy options that focus on individual needs and symptom relief require an assessment of the patient’s medical history to provide the best possible symptom relief [7, 8]. The assessment of patients admitted to a palliative care unit differs considerably from traditional assessments as it mainly focuses on distressing symptoms and psychosocial issues [9–13].

In PC, simple, rapid and feasible measurement instruments are needed to assess individual needs and to evaluate changes. Patients in PC units mostly have a high symptom burden and should therefore not be exposed to time-consuming assessment instruments. A simple risk screening of patients in a PC inpatient setting, which could possibly be integrated into the basic assessment on admission to hospital, is of high importance [14]. Specially developed assessment tools help to document and evaluate the effectiveness of medical, nursing, and therapeutic measures in palliative care units to define and adapt individualized, symptom-oriented treatment strategies for patients [15]. Best practice in PC needs to be continuously adapted to specific patient-centered needs and values [16].

In addition to psychosocial and spiritual support for patients and their caregivers, symptom assessment is a central aim of PC.

A widespread tool is the Edmonton Symptom Assessment System (ESAS) for self-assessment of symptom intensity of nine common symptoms in PC. The ESAS-Revised (ESAS-r), a revised version of the ESAS, has been demonstrated to be more easily understood and preferred by patients due to its clarity and layout [17].

Neither the ESAS nor the ESAS‑r contains an assessment of the social situation of patients, which is an essential part of PC.

The use of an assessment tool must be combined with a careful physical examination and clinical conversation to determine the details of each symptom.

The aim of this study was to implement and evaluate the feasibility of a previously developed structured palliative assessment tool in a rural palliative care unit. Using a numeric rating scale from 0–10, the PERS^2^ON (*P*: pain, E: eating, R: rehabilitation, S^2^: social situation, suffering, O: O_2_, *N*: nausea/emesis) score can be used to explore the patient-reported symptom burden. It was developed for medical and nursing staff in PC [15] but even less specialized PC staff or medical students can use it.

It was developed to provide a rapid, comprehensive picture of symptom burden in palliative care, based on expert consensus from a multidisciplinary palliative care team; however, according to the consensus-based standards for the selection of health measurement instruments (COSMIN) recommendations [21], the PERS^2^ON score has not undergone formal psychometric testing. These criteria are crucial for assessing the responsiveness, validity and reliability of health measurement tools. Therefore, the aim of the current study is to investigate the feasibility of the instrument in a real-world context rather than to validate it.

The PERS^2^ON score aims to include broader domains, such as psychosocial and social components, whereas other tools, such as the ESAS‑r, have been thoroughly validated and focus solely on physical symptoms. Although this broader scope raises questions about conceptual clarity and assessment accuracy, it may also be helpful in guiding holistic palliative care.

## Methods

The study was conducted at the palliative care unit of the Landesklinikum Scheibbs, a rural hospital. The palliative care unit consists of eight beds, this was however reduced to six beds during the whole study period due to the COVID-19 pandemic.

All patients with a malignant disease admitted to the palliative care unit between September 2020 and March 2021 were eligible. Exclusion criteria were a life expectancy on admission of less than 3 weeks according to the attending physician as well as the lack of ability to provide informed consent.

Inclusion criteria required a confirmed diagnosis of malignancy and the ability to give informed consent. There were no restrictions based on age, cancer treatment status, or specific cancer type, although only patients with malignancies were eligible.

Although this study was described as a feasibility study, its design did not fully meet current methodological standards for feasibility research. No formal sample size calculation was performed, which limits the interpretation of inferential statistics such as confidence intervals and *p*-values.

The initial PERS^2^ON score was completed jointly by the treating clinician and the patient on admission, which may have introduced bias. To mitigate this, the day 7 and discharge scores were completed by the patient alone, ensuring independent self-assessment.

### The PERS^2^ON score

The PERS^2^ON score includes the following items:

- **P**ain
- **E**ating (cachexia/loss of appetite/weight loss)
- **R**ehabilitation (physical impairment)
- **S**uffering (anxiety/burden of disease/depression)
- **S**ocial situation (possibility for home care/post-discharge care)
- **O**_2_—dyspnea
- **N**ausea/emesis.

**Fig. 1:**
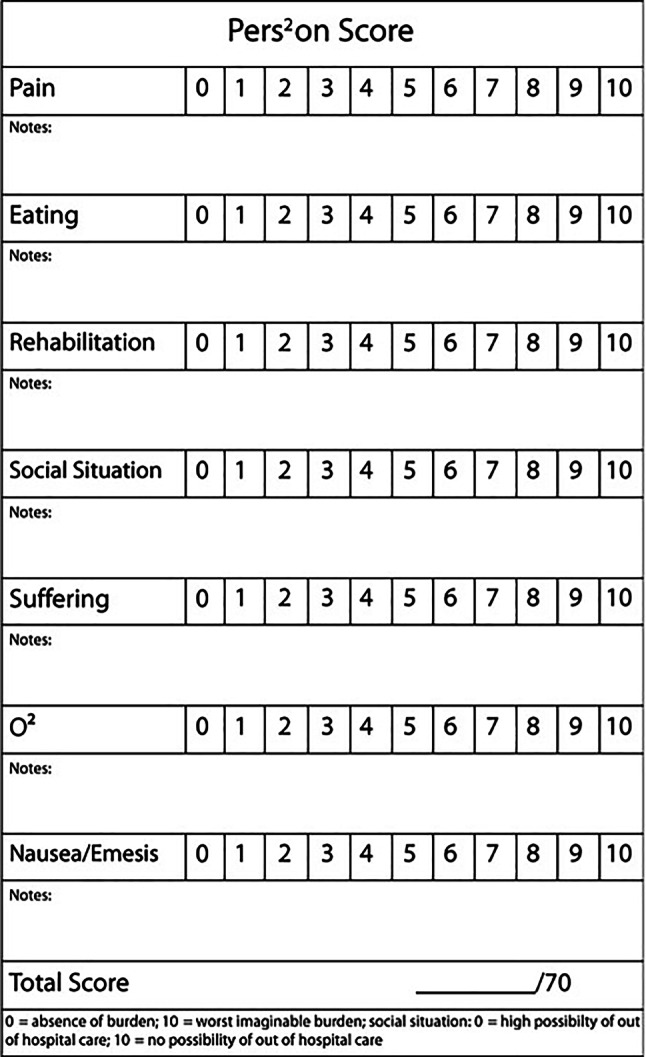
The PERS^2^ON scoring sheet

Each item is rated on a numeric scale between 0 (no burden) and 10 (worst imaginable burden). All seven points are summed resulting in an overall score between 0 and 70, with higher scores representing higher symptom burden. The scoring sheet is provided in Fig. 1.

### Measurement

At inclusion, we recorded patient characteristics including demographics and primary disease. Symptom burden was rated using the PERS^2^ON score at admission, 7 days after administration and on the day of discharge. On the day of admission, the attending palliative care unit physician explained the PERS^2^ON score to the patients, and they completed it together. Nurses or physicians handed the PERS^2^ON scoring sheet to the patients, and they completed it alone 7 days after admission and at the day of discharge.

On admission, the Karnofsky Performance Status Scale (KPS) was assessed as previously published [18].

### Sample size

We did not perform a formal sample size calculation for this study as it was designed as a feasibility study.

## Analysis

We tabulated the overall PERS^2^ON score as well as its seven sub scores by time (admission, 7 days, discharge) using means and standard deviation. The same was done for the Karnofsky Performance Status Scale at admission.

We then calculated absolute and relative frequencies of improvement and deterioration of the PERS^2^ON score as well as absolute differences (including 95% confidence intervals) between the score on admission and the two later measurements. We also compared differences using a paired t‑test with a two-tailed significance level of 0.05.

Statistical analysis was conducted using the Statistical Package for the Social Sciences (SPSS) 20.0 software (SPSS, Chicago, IL, USA).

The study was approved by the local Ethics Committee LAND NÖ GSK (GS4-EK-4/670-2020). The results of this work were previously published as a preprint on Research Square (10.21203/rs.3.rs-3242624/v1).

## Results

### Patient characteristics

**Fig. 2:**
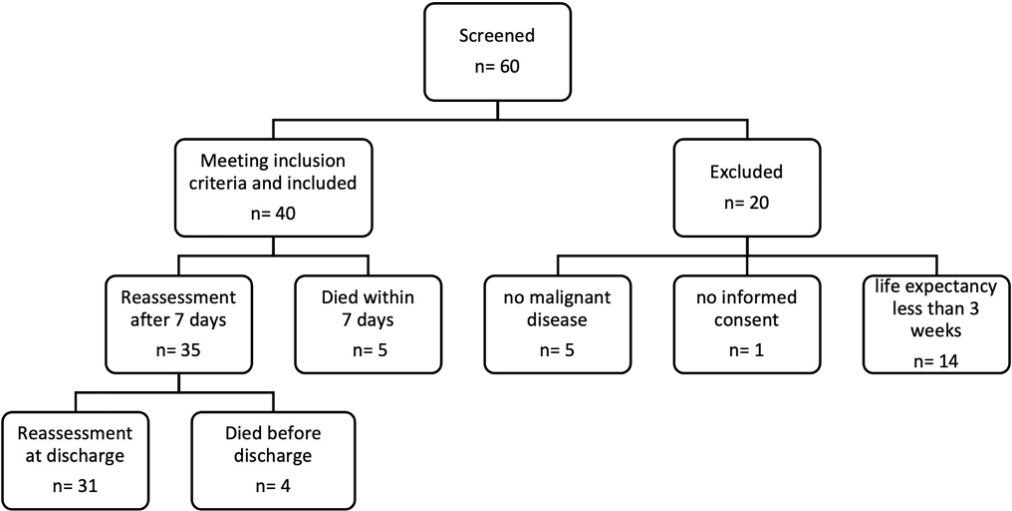
Consort diagram. *n* number

During the study period, a total of 60 patients were admitted to the palliative care unit. As depicted in the CONSORT diagram (Fig. 2), 40 patients met inclusion criteria. Of the remaining 20 patients, 5 lacked an oncological disease, 14 had a life expectancy of less than 3 weeks and 1 patient did not provide consent for inclusion.

All 40 eligible patients were included in the study. Although inclusion required a minimum expected life expectancy of 3 weeks upon admission, nevertheless 9 individuals died during their stay in the palliative care unit, including 5 within the first 7 days.

Of the 40 included patients 27 (68%) were female and overall mean age was 70 years (SD 10). The age range of the participants varied from 36 to 87 years, encompassing the youngest and oldest individuals. The largest age group consisted of 17 individuals (43%) in the 60–69 years category, followed by 10 individuals (25%) in both the 70–79 years and 80–89 years age groups. Among the study participants, 3 individuals (8%) were under 60 years of age upon admission to the palliative care unit.

The average total length of stay was 15 days (SD 9) in the palliative care unit. The minimum length of inpatient admission was 3 days, while the maximum length reached 36 days.

As shown in Table 1, the study participants had 18 different types of cancer. The most common diagnosis was breast cancer with 15% (*n* = 6), followed by lung cancer and pancreatic cancer with 12.5% each (*n* = 5). Other cancer types of the participating patients were rectal cancer with 12.5% (*n* = 5) followed by 7.5% (*n* = 3) cervical cancer and 5% each (*n* = 2) people with ovarian, prostate, and cecal cancer and chronic myeloid leukemia. The remaining nine cancer types are shown in detail in Table 1.

**Table 1:** Patients’ characteristics

	Entire cohort (*n* = 40)	
	*n*	%
Mean age, years (range)	70 (36–87)	–
*Primary tumor*		
Breast cancer	6	15
Lung cancer	5	12.5
Pancreatic cancer	5	12.5
Colorectal cancer	5	12.5
Cervical cancer	3	7.5
Chronic myeloid leukemia (CML)	2	5
Cecal cancer	2	5
Ovarian cancer	2	5
Prostate cancer	2	5
Carcinoma of unknown primary (CUP)	1	2.5
Liver cancer	1	2.5
Lymphoma	1	2.5
Gastric cancer	1	2.5
Melanoma	1	2.5
Multiple myeloma	1	2.5
Renal cell carcinoma	1	2.5
Thyroid cancer	1	2.5

The baseline evaluation using the PERS^2^ON score took approximately 10 min for completion during the initial assessment. On the day of admission, the attending palliative care unit physician explained the score to the patients. Subsequently, 34 patients (85%) required assistance and completed the assessment together with the physician.

The mean baseline PERS^2^ON score was 28 (SD 12) on admission to the palliative care unit. The highest mean sub-scores were observed for rehabilitation (mean 6, SD 3) followed by loss of appetite (mean 5, SD 4) and suffering (mean 5, SD 3). Table 2 lists details on the mean scores of all PERS^2^ON items. The KPS on admission was 53% (SD 13).

**Table 2:** PERS^2^ON score at baseline, 7 days after admission and at the day of discharge

	Baseline		7 days after admission		Day of discharge	
	*n* = 40		*n* = 35		*n* = 31	
	Mean	SD	Mean	SD	Mean	SD
**PERS**^**2**^**ON score**	28	12	22	11	17	10
**P**ain	4	4	3	2	2	2
**E**ating	5	4	4	3	2	3
**R**ehabilitation	6	3	5	2	5	3
**S**uffering	5	3	4	3	3	3
**S**ocial setting	3	3	2	3	1	2
**O**_**2**_	2	3	2	3	2	2
**N**ausea/emesis	5	4	2	2	2	2

### Repeated measurements of the PERS^2^ON score

Completion of the PERS^2^ON score took approximately 5min 7 days after admission and at the day of discharge.

The mean PERS^2^ON score 7 days after admission was 22 (SD 11). The highest sub-scores were observed for potential of rehabilitation (mean 5, SD 2), followed by eating (mean 4, SD 3) and suffering (mean 4, SD 3).

Mean PERS^2^ON score at day of discharge was 17 (SD 10).

Highest sub-scores were observed for the potential of rehabilitation (mean 5, SD 3), followed by suffering (mean 3, SD 3) and eating (mean 2, SD 3).

### Change of PERS^2^ON score

The mean change of the overall PERS^2^ON score within the first 7 days of admission was 7 (95% CI: 3–11). Of the patients 24/32 (75%) presented with an improvement in the PERS^2^ON score; 5/32 (15.6%) patients experienced a deterioration reflected in a higher PERS^2^ON score; 3/32 (9.3%) patients reported no change in PERS^2^ON score.

**Fig. 3:**
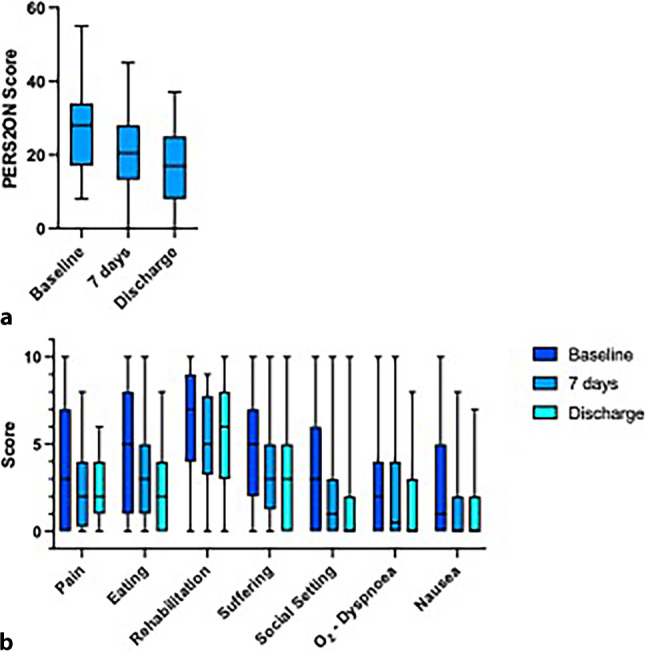
Change in the PERS^2^ON score. **a** Change of the PERS^2^ON score from baseline to 7 days after the admission and day of discharge. **b** Change of each item of the PERS^2^ON score from baseline to 7 days after the admission and day of discharge

Compared to the baseline PERS^2^ON score, the PERS^2^ON score 7 days after admission showed significantly lower values (mean 28, SD 12 vs. mean 21, SD 11, *p* = 0.002). Figure 3 displays the change in the PERS^2^ON score (Fig. 3a) as well as the change in each item (Fig. 3b) from baseline to 7 days after admission.

The mean change of PERS^2^ON score from baseline to the day of discharge was 11 (95% CI: 6–15). Of the patients 12/13 (92%) presented with improvement of PERS^2^ON score from baseline compared to the day of discharge compared to only 1/13 (8%) patient reporting with a decline of PERS^2^ON score.

Compared to the baseline PERS^2^ON score, the PERS^2^ON score on the day of discharge presented with significantly lower values (mean 28, SD 12 vs. mean 17, SD 10, *p* < 0.001). Figure 3 displays the change in the PERS^2^ON score (Fig. 3a) as well as the change in each item (Fig. 3b) from baseline to the day of discharge.

## Discussion

The primary objective of this study was to assess the feasibility of implementing the PERS^2^ON score in a rural palliative care unit setting. Results showed that symptom burden could be captured at multiple time points and that scores decreased over time, indicating clinical responsiveness; however, this study did not assess the psychometric properties of the PERS^2^ON score, such as validity, reliability or sensitivity to change. Future research should include rigorous validation procedures as outlined in the COSMIN guidelines, ideally using multisite, mixed-method approaches.

Getting to know the patient as a “person” is the basis for determining individual distress. In this prospective cohort study, the assessment of the PERS^2^ON score over the stay at the palliative care unit was possible in all patients meeting the inclusion criteria. Whereas most patients needed assistance when completing the score for the first time on admission, all were able to do so unaided at later reassessments.

The mean observed PERS^2^ON score in our cohort was 32, with the highest observed score reaching 54 out of a total of 70 points. These findings emphasize the critical need to address the substantial symptom burden experienced by patients upon admission to a palliative care unit.

Structured assessment and classification of distressing symptoms is the basis for appropriate, individualized symptom management for patients with advanced diseases [19].

The PERS^2^ON score, a simple and easy to use symptom assessment tool, was well accepted by patients and could be completed at all designated time points. Most patients (75%) showed significant improvement after 7 days, particularly in rehabilitation, eating, and alleviating suffering. The score helped guide tailored interventions: physiotherapy for physical impairment, nutritional support for cachexia and weight loss, and psychological or psychiatric care for suffering. These targeted measures were initiated promptly based on individual symptom profiles. The results demonstrate the practical value of the PERS^2^ON score in supporting personalized treatment decisions and improving symptom control in palliative care settings.

Compared to other assessment tools, the PERS^2^ON score also considers the possibility of home care. Social and economic needs of patients can hinder discharge and home care and lead to self-perceived burden as well as stress for caregivers [20–23].

The PERS^2^ON score was used to identify patients with a weak social network for whom outpatient care was unlikely. Thus, a mobile palliative team and family members were involved at an early stage to discuss possible pathways of care with the patients.

The experience of suffering is multidimensional and includes body image, desires, meaning of the illness, relationships, values, and spiritual beliefs. It cannot be classified by symptom assessment alone but requires interaction with the patient’s individual experience [24]. Psychological distress has been reported to decrease with adequate pain relief [25, 26], while mortality rates have been shown to be up to 25% higher in people or their carers who experience depressive symptoms [27–29].

The results of our study reflect that the multidisciplinary and comprehensive approach of a PC team leads to significant symptom relief even in the very short time span of only 7 days, facilitated by the systemic symptom assessment with the PERS^2^ON score. Furthermore, the use of a patient questionnaire is not essential when using the PERS^2^ON score, as it is easy to memorize, which supports its practical application.

Based on the results of this study, the PERS^2^ON score can be effectively used. It provides a quick, efficient, and structured assessment of symptom burden. Its self-explanatory style highlights the potential for its use not only for specialized staff but also for less specialized personnel such as medical students and non-PC professionals. Its applicability extends beyond assessing symptom burden and proved valuable in facilitating a structured medical history-taking process. By incorporating the PERS^2^ON score into clinical practice, diverse healthcare professionals can gather comprehensive information and better understand the multifaceted needs of patients receiving PC. Inconsistencies between patient-reported and clinician-documented symptoms are a common pitfall [30–32], therefore we want to highlight the PERS^2^ON score as a structured, patient-reported assessment that avoids misunderstandings. It proved easy to implement in clinical practice and was appreciated by both clinicians and patients.

This single-center study had a small sample size and potential reporting bias. The PERS^2^ON score was not compared to other validated tools, and spiritual needs were not systematically assessed. Conceptually, the score combines diverse constructs (e.g., suffering, physical symptoms, social factors), which limits its clarity and applicability as a standardized outcome measure. Moreover, only cancer patients were included, restricting generalizability to the broader palliative population. Future studies should involve patients with non-malignant conditions and include stakeholder input to improve conceptual validity and ensure the score reflects a needs-based approach.

## Conclusion

The evaluation and testing of the PERS^2^ON score demonstrated its suitability as a user-friendly symptom assessment tool that can be integrated into the routine practice of rural palliative care units. By providing an accessible scale, it serves as a valuable guide for treatment decisions and contributes to prompt symptom alleviation. The implementation of the PERS^2^ON score has great potential to enhance the quality of care and facilitate rapid improvements in symptom management within PC settings.
